# Endobronchial ultrasound transbronchial needle aspiration in the evaluation of the mediastinum lymph nodes: safety evaluation

**DOI:** 10.1186/1939-4551-8-S1-A78

**Published:** 2015-04-08

**Authors:** Kostantinos Syrigos, Ioannis Kokkonouzis, Ioannis Lampaditis, Elias Kainis, Andriani Charpidou

**Affiliations:** 1 Sotiria General Hospital, Athens School of Medicine, Greece

## Background

Evaluation of mediastinal lymph nodes is of paramount importance for accurate staging in lung cancer and often dictates optimal treatment. Mediastinoscopy is considered the "gold standard" for sampling lymph nodes, but it is an invasive, costly technique that requires general anesthesia. Newer staging techniques have emerged. Endobronchial ultrasound-guided transbronchial needle aspiration (EBUS-TBNA) is a minimally invasive technique that permits accurate and adequate sampling of mediastinal and hilar lymph nodes under direct, real-time visualization of lesions. The aim of this review is to evaluate the safety profile of this increasingly performed procedure.

## Methods

A detailed search was conducted through bibliographic databases (Pubmed Central, Medline) using combinations of the terms "complications or adverse events", "endobronchial ultrasound transbronchial needle aspiration" and "medastinum or mediastinal lymph nodes".

## Results

Fifty-five studies were retrieved reporting the presence or absence of complications following EBUS-TBNA. Eighteen of these focused specifically on safety issues. Complication rates ranged from 0.15% to 1.44% in meta-analyses, large retrospective reviews and nationwide surveys. The results of smaller studies were heterogeneous, but overall few adverse events have been reported. Case reports have also been included in the review.

## Conclusions

So far, based on data from well-structured studies, originating from experienced medical centers, EBUS-TBNA is a safe technique with rare complications. On the other hand, some studies have raised issues mainly on the infectious and traumatic complications of this technology, and although data are not sufficient, these issues must be put under consideration. Nevertheless, overall EBUS remains a safe procedure.

